# Large-Scale Assessment of Health-Related Physical Fitness in French Older Adults: Feasibility and Validity

**DOI:** 10.3389/fpubh.2020.487308

**Published:** 2020-12-17

**Authors:** Damien Mack-Inocentio, Mehdi Menai, Eric Doré, Bastien Doreau, Camille Gaillard, Julien Finaud, Bruno Pereira, Pascale Duché

**Affiliations:** ^1^Université Clermont Auvergne, Laboratoire des Adaptations Métaboliques à l'Exercice en conditions Physiologiques et Pathologiques (AME2P, EA 3533), Clermont-Ferrand, France; ^2^Centre de Recherche en Nutrition Humaine d'Auvergne, Inra, Clermont-Ferrand, France; ^3^Université Paris 13, Sorbonne Paris Cité - EREN (Equipe de Recherche en Epidémiologie Nutritionnelle), U1153 Inserm, Inra, Cnam, Centre de Recherche en Epidémiologie et Biostatistiques; CRNH IdF, Bobigny, France; ^4^Université Clermont Auvergne, CNRS, Laboratoire d'Informatique, de Modélisation et d'Optimisation des Systèmes, Clermont-Ferrand, France; ^5^Association Sportive Montferrandaise, Clermont-Ferrand, France; ^6^CHU Clermont-Ferrand, Unité de Biostatistiques (DRCI), Clermont-Ferrand, France; ^7^Université de Toulon, Laboratoire Impact de l'Activité Physique sur la Santé (IAPS), Toulon, France

**Keywords:** validity, physical activity, older people, test battery, physical fitness

## Abstract

**Objectives:** This study aims to assess the validity, internal consistency, implementation, and feasibility of a sequence of tests, the Vitality Test Battery, designed to measure physical fitness, at a large scale in French older adults.

**Methods:** A total of 528 volunteers (age ≥60 years) took the battery of 10 tests: 6-min walk, trunk strength, hand grip strength, medicine ball throwing, 30-s chair stand, flexibility, balance, plate tapping, ruler drop, and dual task.

**Results:** Internal consistency was high, with the Cronbach alpha coefficients at around 0.77, explaining 64% of the variance. The test–retest correlations (0.3–0.6) between the items were acceptable and displayed an internal consistency property. Although five components explained 65% of the variance, all the items were kept because their eigenvalues were near to 0.9. External consistency was validated by a significant decrease in fitness scores (*p* < 0.001) with age and body mass index.

**Discussion:** The Vitality Test Battery is a safe, valid tool for assessing physical fitness in persons aged over 60 years.

## Introduction

The proportion of older people is increasing worldwide. In 2013, 23.8% of the French adult population were of age 60 years or older, and by 2070, this proportion could reach 34.5% (1). Aging populations present economic and societal challenges (2, 3). Among these, maintaining good levels of physical fitness in the older population helps preserve autonomy and functional abilities (4, 5). Physical fitness improves healthy aging (6, 7) and helps in successful aging (7, 8). However, physical activity decreases with age in both men and women, adversely affecting their physical fitness through diminished muscle strength and endurance and changes in body composition (9).

Physical fitness is defined by physical and physiological characteristics. Its components may vary according to the definition used. The main ones are usually taken to be body composition, cardiorespiratory endurance, muscular strength and endurance, and flexibility and balance (10). Physical fitness and its components have been shown to be associated with various health outcomes, including healthy aging (6, 7). Various tests can assess physical fitness, including specific sub-population tests (e.g., seniors) (11). However, most of these tests are not suitable for rapidly assessing large groups. It has long been recommended that data from valid, reliable measures of fitness (12) be used in physical fitness assessments to measure and promote physical activity and health (13). However, attendant time, cost, and expertise requirements may limit the implementation on a large scale (14). The challenge is thus to simplify the evaluation process by ensuring the accuracy and the reliability of the physical fitness assessment in all components.

Given the countless benefits of physical activity for older persons, significant emphasis should be placed on promoting their physical activity to maintain or improve physical fitness. All stakeholders, including policy makers, local and national public services, and health care providers need to evaluate the physical fitness of the population to establish clear and effective fitness goals. Developing easy-to-use tools assessing fitness quickly and safely is thus important, particularly for prescribing adapted and personalized physical fitness-related programs on a large scale.

Furthermore, since 2012, in France, the Ministry of Sports and Ministry of Health have sought to implement measures to promote and develop the practice of physical activity (especially leisure-time physical activity) for people with chronic non-communicable diseases (diabetes, hypertension, cancer, *etc*.) by the “Sport, Health and Welfare” Plan. In 2016, a law allowing general practitioners to prescribe physical activity appropriate to a patient's health condition, physical abilities, and medical risk was passed. To help establish protocols of physical activity adapted to healthy persons and to patients according to their health status, an expert panel of eight members (representatives of sport science, public health, IT engineering, and exercise physiology) was set up to recommend preexisting valid and relevant tests. To meet all the constraints while adhering to the narrow specifications given by general practitioners, sport associations, and public health institutions, the Vitality Test Battery is designed to:

- evaluate the physical fitness of a group of individuals in a short time,
- not require a large number of supervisors,
- be implemented in a restricted space indoors or outdoors, and
- guarantee the accuracy and validity of the tests and their results.

Accordingly, in a population of healthy older persons and patients without severe motor limitations, the study sought to assess (i) the validity and internal consistency of the Vitality Test Battery and (ii) the implementation and feasibility of the test battery to quickly assess the physical fitness of seniors.

## Methods

### Design

The study was performed in two steps:

- Day 1: The test battery was administered with three different strategies: (i) one volunteer with one qualified supervisor, (ii) 10 volunteers with one supervisor, and (iii) 10 volunteers with two supervisors. At the end of day 1, the expert panel considered the third condition to be best for the subsequent days. The results of all the Vitality Test Battery tests are shown in Table 1.
- Days 2 and 3: Physical fitness during a public event in a gym was assessed. Groups of 10 to 12 persons with two supervisors, with a start every 15 min, were tested, totaling ~270 persons per day (for a total of 10 supervisors).

**Table 1:** Test time by the number of supervisors and participants (test time = instructions + test + interpretation of results).

**Vitality test battery**	**One participant, one supervisor**	**10 participants, one supervisor**	**10 participants, two supervisors**
6-min walk test	8 min	11min	9 min 30 s
Hand grip strength test	1min 30 s	4 min	3 min
Trunk strength test	2 min	4 min	3 min
Medicine ball throwing test	2 min	6 min	5 min 30 s
30-s chair stand test	1 min 30 s	3 min 30 s	2 min 30 s
Flexibility test	1 min 30 s	4 min	3 min
Balance test	2 min 30 s	3 min	2 min 30 s
Plate tapping test	1 min 30 s	4 min	3 min
Ruler drop test	1 min 30 s	4 min 30 s	3 min 30 s
Dual task test	1 min 30 s	7 min 30 s	6 min 30 s
Interpretation of results	4 min	8 min	6 min
Total time	27 min 30 s	59 min 30 s	48 min

### Selection of Study Sample

A health insurance company invited 1,500 local retirees to participate in fitness evaluation days in Clermont-Ferrand (France) between November 2016 and November 2017. The first 550 respondents were selected. Finally, a group of 528 persons volunteered to take part in the study.

Persons under 60 years of age or living in institutions with a physical or mental illness that limited their participation in fitness tests or their ability to answer questionnaires were excluded. All procedures involving human subjects were approved by the French data protection authority, Commission Nationale de l'Informatique et des Libertés (CNIL), and the University Review Board. In accordance with the Declaration of Helsinki, written informed consent was obtained from each participant prior to inclusion and participation in the tests.

### Protocol

Each participant filled in a self-administered tablet-based questionnaire before the Vitality Test Battery was implemented. Data included birth date, gender, and personal information (address, phone, etc.).

At 1 week before the Vitality Test Battery day, all the participants completed the first part of a questionnaire on physical activity capacities, the “Get Active Questionnaire” (GAQ), designed to screen the participants about their physical activities to ensure that they can engage in exercise safely. The GAQ was developed by an expert panel from the Canadian Society for Exercise Physiology (http://www.csep.ca/home) and tested by Petrella et al. (15). The GAQ was used here to support pre-participation screening in physical activities and to help identify risk factors to be considered before engaging in physical activity. All participants found at risk were excluded.

Before taking the 10 tests, all the participants did a standardized warm-up for 10 min. The warm-up included articular and muscular mobilization of the whole body, a stretching phase, and a low-intensity walking phase.

### Computation

Inherent computation from the different components of the Vitality Test Battery was carried out in real time with a tablet computer (Pro Slate 10 EE Android). A specific software was developed by our partner, the Laboratory of Computing, Modeling and Optimization of Systems. This setup could record from several groups in the same time period with staggered starts. At the end, each participant received a results booklet with interpretation and advice from the supervisors.

#### Vitality Test Battery

The Vitality Test Battery consisted of 10 conventional physical tests to evaluate the six components of fitness-related health: body composition, cardiorespiratory endurance, muscular endurance and strength, flexibility, balance, and motor coordination.

### Body Composition

With the participants standing with minimal clothing, body mass was measured with a digital scale. While they were in barefoot standing position, height was measured using a calibrated stadiometer. Body mass index (BMI) was calculated as body weight divided by height squared (kg/m^2^).

### Cardiorespiratory Endurance

The six-min walk test (6MWT) assessed the submaximal level of functional cardiorespiratory capacity. The 6MWT is a simple, practical test to assess activities of daily living (16). Walking is an activity performed daily by all healthy people and patients without severe motor limitations. The 6MWT could be performed indoors and outdoors. The turnaround points were marked with a cone (e.g., an orange traffic cone). A line indicating the beginning and the end of each 30 m lap was marked on the floor with brightly colored tape. The participants were instructed to try to cover as much distance as possible within 6 min only by walking. Incentive encouragements were given by supervisors every 30 s. The distance that a participant could quickly walk on a flat, hard surface in a period of 6 min was recorded. The validity and the reliability of the 6MWT in older adults were verified by Rikli and Jones (17).

### Muscular Endurance and Strength

General muscular capacity was evaluated with five different tests:

1. The trunk strength test (abdominal muscular endurance): The participants had to perform three sets of five different abdominal trials used to assess abdominal strength. The sets were of increasing difficulty. For the first five sit-ups, the participants had to reach the mid-patella with the fingertips of both hands from a straight lying position, keeping their arms straight and their palms resting on thighs. For the second five sit-ups, with arms folded over the chest, it was aimed to reach the thighs with both elbows. For the last five sit-ups, it was aimed to reach the thighs with the elbows, touching the back of the earlobes with the fingertips. The number of bouts performed was recorded. This validated test is part of the Eurofit Test Battery for Adults (18).
2. The hand grip strength test (HGS test): The participants had to use a hand dynamometer that assessed gripping force. HGS was measured with a hand-held dynamometer (Takeï TK200, measuring between 5 and 100 kg, in increments of 0.1 kg). HGS was evaluated only in the dominant hand. The subjects were encouraged to perform two maximal contractions at a 10-s interval, and the best value was recorded. The test was proposed and validated by the Eurofit Battery Test for Adults (18). It is a valid measurement in healthy female and male subjects (19).
3. The medicine ball throwing test: The participants had to sit with their back against a wall to assess the explosive force of the upper limbs. This test is the measurement of the farthest distance a subject can throw a medicine ball with both hands while sitting. Each volunteer performed two throws of a medicine ball weighing 2 kg for women and 3 kg for men. The best distance was recorded. This test is a highly reliable test of upper body power (20).
4. The 30-s chair stand test (30CST): The participants were asked to rise to a full stand from a fully seated position and complete as many full stands as possible in 30 s. The arms must be crossed during the test. The number of full stands was recorded to assess lower body strength. This validated test is part of the Eurofit Test Battery for Adults (18). It provides a reasonably reliable and valid indicator of lower body strength (21).

### Flexibility

Flexibility was assessed using the seated flexibility test. The distance between the fingers and the toes was measured. The distance was positive when the fingers went beyond the feet and negative when the fingers did not reach the feet. A measuring box was used. This validated test is part of the Eurofit Test Battery for Adults (18) and produces reasonably accurate and stable measures of hamstring flexibility (22).

### Balance

The balance test of Bohannon et al. (23) (30 s with eyes open and closed on a chosen leg) was used. Its target is to reach 30 s maximum without loss of balance and/or support of the free leg (23). The longest times achieved for each balancing activity were recorded, and the following three results were analyzed: eyes-open time, eyes-closed time, and the difference between them (Δ).

### Motor Coordination

Three fitness tests were used to evaluate motor skills:

1. The plate tapping test: The aim of this test is to assess the coordination of arm activity and speed by measuring the time for the dominant hand to touch two disc 80 cm apart 25 times while the other hand is fixed between the two disc. The best time to make 25 back-and-forth movements is recorded. This validated test is part of the Eurofit Test Battery for Adults (18).
2. The ruler drop test of Mackenzie (24), in which the subject must catch as quickly as possible a ruler 40 cm long dropped by the examiner, evaluates reaction time.
3. The dual task test of Lundin-Olsson et al. (25) assesses coordination by measuring the time difference between a 10-m simple brisk walk and a 10-m brisk walk with a constraint (holding a ball balanced on a plate without dropping it). Dual task tests typically require the participants to divide their attention and concurrently execute two different tasks. Inability to perform two or more tasks simultaneously (multi- or dual-tasking) is regarded as an indicator of a higher fall risk (26).

All the tests were performed twice, except for the 6-min walk test, and the higher score was retained (25). The order of the tests was as follows: 6-min walk test (m), trunk strength test (*n*), hand grip strength test (N/kg), medicine ball throwing test (m), 30-s chair stand test (*n*), flexibility test (cm), balance test (Δs), plate tapping test (s), ruler drop test (cm), and dual task test (Δs).

### Statistical Analysis

Besides the usual descriptive statistics to characterize the population (continuous data were expressed as mean ± standard deviation), psychometric properties were evaluated according to COSMIN guidelines (27).

Internal consistency property was measured in a variety of ways, Cronbach's alpha (for all items and for one measure if a single item was removed), correlations between an item and the remaining items in the measure (called corrected item-scale correlations), the average inter-item correlation, the range of inter-item correlations, and the individual inter-item correlations of the scale. Cronbach's α calculated to evaluate internal consistency property tells us the extent to which items in the questionnaire are consistent and measure attributes of a single concept. Values closer to one indicate a higher internal consistency, and values closer to zero indicate a lower internal consistency. For a good scale, the average inter-item correlations should be between 0.15 and 0.50. Item means, standard errors, and inter-item total correlations were calculated.

When internal consistency is relevant, principal component analysis (PCA) or factor analysis should be applied to determine whether the items form only one overall scale. Factor analysis was applied to provide empirical support for the dimensionality of the questionnaire. To analyze relationships between tests and then identify groups of tests with similar characteristics, a principal component analysis was performed. The number of factors was chosen according to the usual recommendations: Kaiser criteria, plot of eigenvalues, and the proportion of variance expressed by the principal component.

Finally, to evaluate construct validity and test hypotheses, a score of the first component was established as a linear combination of the original variables to generate the first principal component, i.e., fitness score was calculated so that it accounted for the greatest possible variance in the data. This score was compared to the participants' age and BMI using a correlation coefficient (Pearson or Spearman according to statistical distribution) for analysis between quantitative parameters, using ANOVA or Kruskal–Wallis test when appropriate (omnibus *p* < 0.05), and by a *post hoc* test for multiple comparisons (Tukey–Kramer or Dunn) for categorical parameters (age and BMI categorized according to statistical distribution and clinical relevance). The comparisons between women and men were made using multiple linear regression, taking into account the BMI adjustment. The normality of residuals from these models was studied with the Shapiro–Wilk test. When appropriate, a logarithmic transformation of the dependent variable (each item) was applied to obtain normality.

Statistical analyses were performed using Stata software, version 13 (StataCorp, College Station, TX, USA). An α-level of <0.05 was used to determine statistical significance in each of the analyses.

## Results

### Sample Characteristics

The sample comprised 528 participants (53% women and 47% men) (Table 2). There was no difference between women and men for age (67.5 ± 5.4 and 67.6 ± 5.5 years, respectively; *p* = 0.924). BMI was significantly higher in men than in women (25.9 ± 3.3 vs. 24.5 ± 4.3 kg/m^2^, *p* < 0.001).

**Table 2:** Characteristics of the participants.

	**Women (*****n*** **=** **281)**			**Men (*****n*** **=** **247)**			***p*-value**
	**Mean ± SD**	**Min**	**Max**	**Mean ± SD**	**Min**	**Max**	
Age (years)	67.5 ± 5.4	60.0	88.0	67.6 ± 5.5	60.0	86.0	Ns
Height (cm)	161.1 ± 6.1	145.0	178.0	173.8 ± 6.5	154.0	190.0	<0.001
Body mass (kg)	63.7 ± 12.2	38.0	111.0	78.3 ± 12.3	51.0	123.0	<0.001
Body mass index (kg/m^2^)	24.5 ± 4.3	15.6	44.1	25.9 ± 3.3	18.5	38.3	<0.001

### Implementation of the Vitality Test Battery and Results for Women and Men

The Vitality Test Battery results are shown in Table 3. The most efficient organization used during the test day was the one with groups of 10 participants and two supervisors. Table 3 shows the results of the Vitality Test Battery for women and men. Except for the flexibility test, all physical fitness parameters were lower in women than in men.

**Table 3:** Results and comparison of physical fitness parameters from the tests in the Vitality Test Battery between women and men.

	**Overall**	**Women**	**Men**	***p*-value men vs. women**
6-min walk test (m)	571.0 ± 81.4	557.0 ± 79.5	590.9 ± 80.0	<0.001
Trunk strength test (*n*)	10.0 ± 5.4	9.1 ± 5.4	11.3 ± 5.1	<0.001
Hand grip strength test (N/kg)	4.6 ± 1.1	4.0 ± 0.9	5.4 ± 1.0	<0.001
Medicine ball throwing test (m)	3.2 ± 0.7	2.8 ± 0.5	3.7 ± 0.6	<0.001
30-s chair stand test (*n*)	15.1 ± 3.2	14.7 ± 3.0	15.6 ± 3.4	<0.001
Flexibility test (cm)	−0.2 ± 10.2	3.3 ± 8.0	−5.2 ± 10.9	0.004
Balance test (Δs)	16.0 ± 9.2	15.3 ± 9.3	17.1 ± 9.0	<0.001
Plate tapping test (s)	13.4 ± 3.4	14.0 ± 3.7	12.6 ± 2.8	0.015
Ruler drop test (cm)	21.8 ± 7.0	22.4 ± 7.1	20.8 ± 6.8	<0.001
Dual task test (Δs)	2.1 ± 1.8	2.5 ± 2.0	1.7 ± 1.5	<0.001

### Internal Consistency Property

The interpretation of the balance test results was examined to select the best balance test modality. The Cronbach's alpha coefficients were 0.77 for the overall population, explaining 64% of the variance, and 0.70 for women and 0.72 for men for the interpretation of the delta balance test result with eyes open and eyes closed (EO–EC). For the eyes-open balance test, the Cronbach's alpha values were 0.76 for the overall population, 0.71 for women, and 0.70 for men. For the eyes-closed balance test, the Cronbach's alpha values were 0.76 for the overall population and 0.70 for women and men. The highest Cronbach's alpha value was obtained when the delta of the balance test results was used.

The internal consistency of the Vitality Test Battery was calculated with the balance test delta. Cronbach's alpha coefficient including 10 items was 0.77 for the overall population, ranging from 0.73 to 0.78 (Table 4). The coefficient decreased and was 0.70 for women (ranging from 0.65 to 0.71, Table 4) and 0.72 for men (ranging from 0.68 to 0.73, Table 4). The correlations between the items were acceptable and presented good reliability and internal consistency. For gestural and dual task tests, correlations of <0.30 were found.

**Table 4:** Internal consistency of the vitality battery test (Cronbach alpha) for the overall population, for women, and for men.

	**Item–test correlation**	**Item–rest correlation**	**Average inter-item correlation**	**Cronbach alpha**
**Overall**				
6-min walk test	0.63	0.51	0.25	0.75
Trunk strength test	0.59	0.46	0.25	0.75
Hand grip strength test	0.7	0.59	0.23	0.73
Medicine ball throwing test	0.65	0.52	0.24	0.74
30-s chair stand test	0.58	0.45	0.25	0.75
Flexibility test	0.52	0.38	0.26	0.76
Balance test	0.57	0.43	0.26	0.76
Plate tapping test	0.44	0.29	0.28	0.77
Ruler drop test	0.64	0.52	0.24	0.74
Dual task test	0.41	0.25	0.28	0.78
Test scale			0.25	0.77
**Women**				
6-min walk test	0.63	0.49	0.2	0.65
Trunk strength test	0.54	0.39	0.19	0.67
Hand grip strength test	0.61	0.46	0.2	0.66
Medicine ball throwing test	0.49	0.32	0.21	0.68
30-s chair stand test	0.6	0.46	0.2	0.66
Flexibility test	0.55	0.38	0.21	0.67
Balance test	0.47	0.3	0.19	0.69
Plate tapping test	0.43	0.25	0.21	0.7
Ruler drop test	0.53	0.37	0.21	0.67
Dual task test	0.34	0.15	0.23	0.71
Test scale			0.19	0.7
**Men**				
6-min walk test	0.59	0.45	0.2	0.69
Trunk strength test	0.6	0.46	0.19	0.68
Hand grip strength test	0.56	0.41	0.2	0.69
Medicine ball throwing test	0.51	0.35	0.21	0.7
30-s chair stand test	0.55	0.4	0.2	0.69
Flexibility test	0.52	0.37	0.21	0.7
Balance test	0.62	0.48	0.19	0.68
Plate tapping test	0.48	0.32	0.21	0.71
Ruler drop test	0.51	0.36	0.21	0.7
Dual task test	0.38	0.19	0.23	0.73
Test scale			0.2	0.72

### Principal Component Analysis to Determine a Fitness Score

A principal component analysis revealed that five components explained a large proportion of the variance: 65% for the whole population (60% in women and 62% in men) (Table 5). The other five tests were distributed over the three other components. All the components were retained because their eigenvalues were near to 0.90. The score of the first component was established as a linear combination of the original variables to generate the first principal component, i.e., fitness score was calculated so that it accounted for the greatest possible variance in the data set.

**Table 5:** Eigenvalues and proportion of variation explained by the principal component analysis for the overall population, women, and men.

**Principal component**	**Eigenvalue**	**Difference**	**Proportion**	**Cumulative**
**Total sample**				
1 (6-min walk test−6MWT, trunk strength test—TST, hand grip strength test—HGST, medicine ball throwing test—MBTT, ruler drop test—RDT)	3.36	2.16	0.34	0.34
2 (balance test—BT, DTT)	1.20	0.21	0.12	0.46
3 (30-s chair stand test−30sCST, flexibility test—FT)	0.99	0.08	0.10	0.55
4 (plate tapping test—PTT)	0.91	0.10	0.09	0.65
**Women**				
1 (6MWT, TST, HGST, 30sCST, FT)	2.84	1.53	0.28	0.28
2 (BT, DTT)	1.31	0.36	0.13	0.42
3 (MBTT, PTT)	0.95	0.03	0.10	0.51
4 (RDT)	0.92	0.06	0.09	0.60
**Men**				
1 (6MWT, TST, HGST, 30sCST, FT)	2.75	1.52	0.28	0.28
2 (BT, DTT)	1.24	0.10	0.12	0.40
3 (MBTT, PTT)	1.14	0.13	0.11	0.51
4 (RDT)	1.02	0.11	0.10	0.62

### Construct Validity and Hypothesis Testing

**Figure 1:**
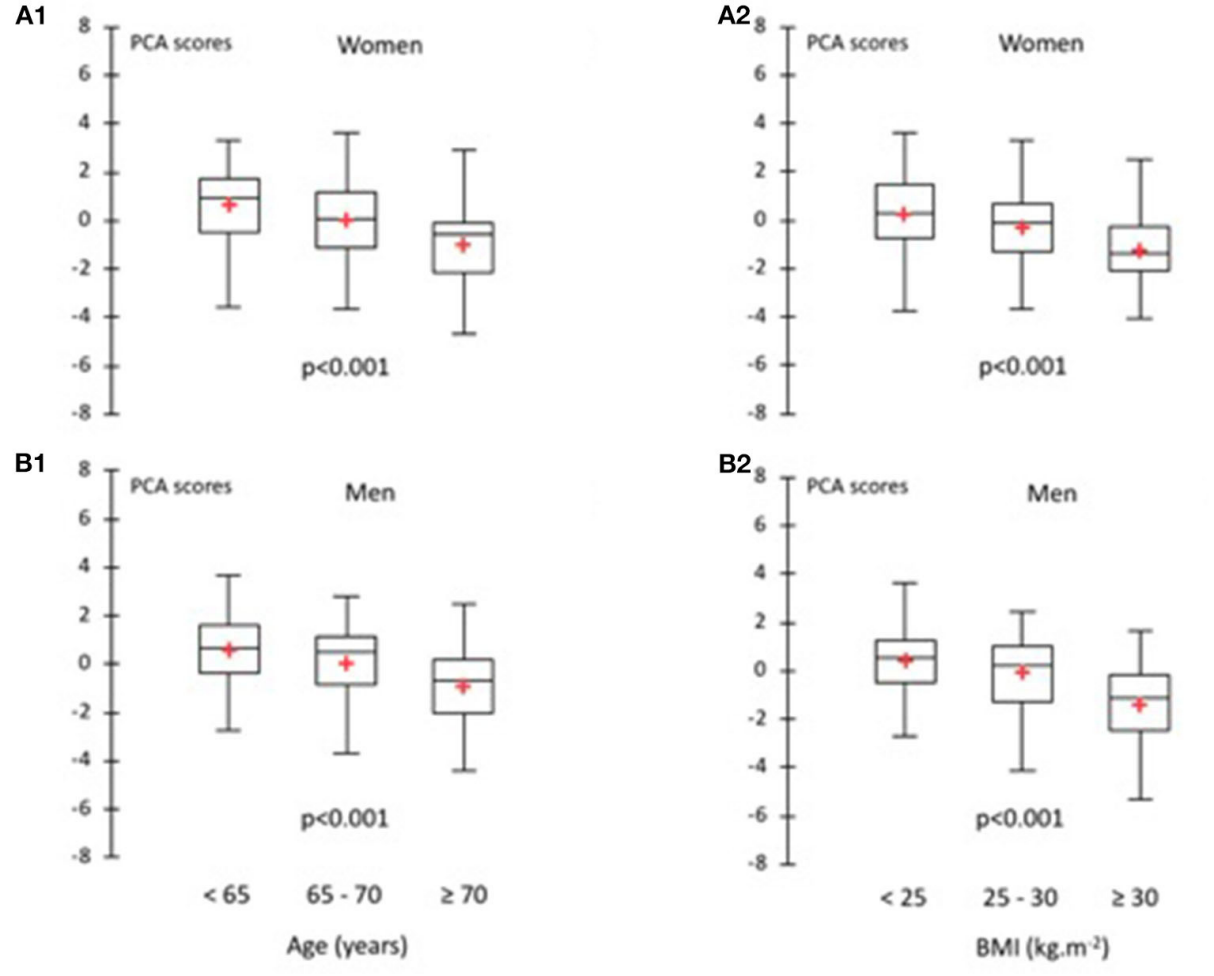
Relationship between physical fitness scores and age for women **(A1)** and men **(B1)** and body mass index for women **(A2)** and men **(B2)**.

Table 6 shows the relationships between fitness score and each test, age (0.39 for women and 0.37 for men), and BMI (0.27 for women and 0.30 for men) using correlation coefficients. This analysis was performed separately on women and men. Significant weak to moderate correlations with each test were observed for each sex group. The physical fitness score calculated from PCA decreased significantly (*p* < 0.001) with age and BMI in both women and men (Figure 1).

**Table 6:** Correlations between fitness score and each test, age, and body mass index for women and men.

	**Women**	**Men**
6-min walk test	0.68^*^	0.58^*^
Trunk strength test	0.59^*^	0.64^*^
Hand grip strength test	0.67^*^	0.58^*^
Medicine ball throwing test	0.39^*^	0.36^*^
30-s chair stand test	0.58^*^	0.59^*^
Flexibility test	0.53^*^	0.43^*^
Balance test	0.36^*^	0.43^*^
Plate tapping test	0.56^*^	0.47^*^
Ruler drop test	0.20^*^	0.23^*^
Dual task test	0.43^*^	0.59^*^
Age	0.39^*^	0.37^*^
BMI	0.27^*^	0.30^*^

* *p < 0.05*.

## Discussion

The main results suggest that the Vitality Test Battery is a valid and easy tool to assess physical fitness in older men and women. Testing groups of 10 persons with two supervisors can take 48 min. The feasibility criteria are therefore met. The Cronbach's alpha coefficients were acceptable, ranging from 0.70 to 0.80. The test–retest correlations between the items were also acceptable, with most of the fitness tests ranging between 0.30 and 0.60. The Vitality Test Battery can thus be used to assess the physical condition of senior men and women and the effects of aging.

The Vitality Test Battery was developed to assess physical fitness outside a laboratory, seeking to provide patients with tailored information on their fitness level and possible goals. It is a simple, inexpensive tool that can be used for prevention or for physical fitness research. It requires space, equipment to perform the tests, and trained supervisors.

According to other studies, the present results show differences between men and women, for the walking test (17, 28–30), the abdominal test (18), the grip test (18), the sit/stand test (21, 31), the flexibility test (22), and the plate tapping test (18). The other tests were new, so our results could not be compared with others.

According to Nunnally and Bernstein (32), “typically, the item–test correlations should be roughly the same for all items. Item–test correlations may not be adequate to detect items that fit poorly because these items may distort the scale. Accordingly, it may be more useful to consider item–rest correlations, that is, the correlation between an item and the scale that is formed by all other items” (32). In the present study, item–rest correlations exceeded 0.20 for all items except the dual task test. According to Briggs and Cheek (33), to obtain an optimal level of homogeneity, the 0.20–0.40 range was acceptable (33). Our results for inter-item correlation (0.19–0.23) were acceptable considering previous guidance (34). The means of Cronbach's alpha were 0.77 for the total population (0.72 for men and 0.70 for women), which is also acceptable (35). The present findings suggest that the Vitality Test Battery is a valid tool for measuring physical fitness in seniors.

There are several balance tests used in the literature, such as the flamingo balance test (EC) (36), the unipedal stance test (EO and EC) (37), single limb stance times (EO) (38), and one-legged timed balance tests (EO and EC). These various protocols show that there are different methods to assess balance. The results of this study reveal that the most efficient method of interpreting the results would be the (EO–EC) delta according to Cronbach values.

The relationships between fitness score and each test, age, and BMI present significant correlations with all tests (*p* < 0.05). Compared with the results of Rikli and Jones (21), which examined the validity of a functional fitness test in the elderly, the present study showed a significant difference (*p* < 0.05) between fitness test performance between age groups 60–69, 70–79, and 80–89 for all the tests in the battery. In the study of Vagetti et al. (39), the elderly women classified as overweight and obese had lower functional fitness scores compared with the elderly women within the normal weight range (39). According to the results of Vagetti's study, our data showed an association between BMI and functional fitness in the elderly participants. Each of the tests is therefore a significant discriminator between age groups and BMI groups. The results obtained in this study confirm the sensitivity of the Vitality Test Battery for evaluating the performance and the functional capacity of persons of different age and body mass index.

The limitations in this cross-sectional study include, firstly, the use of a convenient sample of healthy older community dwellers whose results may not be generalizable to older adults with limited physical abilities. Secondly, not all the fitness tests were validated tests.

## Conclusion

According to Cronbach's α coefficient, the Vitality Test Battery is a reliable and valid tool for assessing physical fitness in the population older than 60 years. The present findings support the use of the Vitality Test Battery to assess fitness in a group of people in a short time and show that this test battery can assess the performance and the functional capacity of persons according to their BMI and age. Finally, this Vitality Test Battery validation process establishes the validity and the internal consistency for its application so that it may be used in practice. These results are useful for assessing physical fitness and promoting physical activity in older persons as these tests are appropriate for this population. A validated test battery is also essential as a starting and ending point for physical activity recovery programs, especially for older persons.

## Data Availability Statement

The datasets generated for this study are available on request to the corresponding author.

## Ethics Statement

The studies involving human participants were reviewed and approved by Commission Nationale de l'Informatique et des Libertés (CNIL) and the University Local Board approved the study. The patients/participants provided their written informed consent to participate in this study.

## Author Contributions

DM-I, MM, and PD wrote the manuscript. PD drafted the study design and supervised the project. DM-I, BD, CG, and JF conducted data acquisition and analysis. BP performed the statistical analysis. All the authors contributed to manuscript revision and read and approved the submitted version.

## Conflict of Interest

The authors declare that the research was conducted in the absence of any commercial or financial relationships that could be construed as a potential conflict of interest.
